# Supplementary material for: H7N9 avian influenza virus infection in men is associated with testosterone depletion
Source: Nat Commun. 2022 Nov 14;13:6936. doi: 10.1038/s41467-022-34500-5 (PMC9662777; doi:10.1038/s41467-022-34500-5)
Supplement: Supplementary file 1 — Supplementary Information [file 41467_2022_34500_MOESM1_ESM.pdf]

Supplementary information

H7N9 avian influenza virus infection in men is associated with testosterone depletion

Tian Bai, Yongkun Chen, Sebastian Beck, Stephanie Stanelle-Bertram, Nancy Kouassi Mounogou, Tao Chen, Jie Dong, Bettina Schneider, Tingting Jia, Jing Yang, Lijie Wang, Andreas Meinhardt, Antonia Zapf, Lothar Kreienbrock, Dayan Wang, Yuelong Shu, Gülsah Gabriel

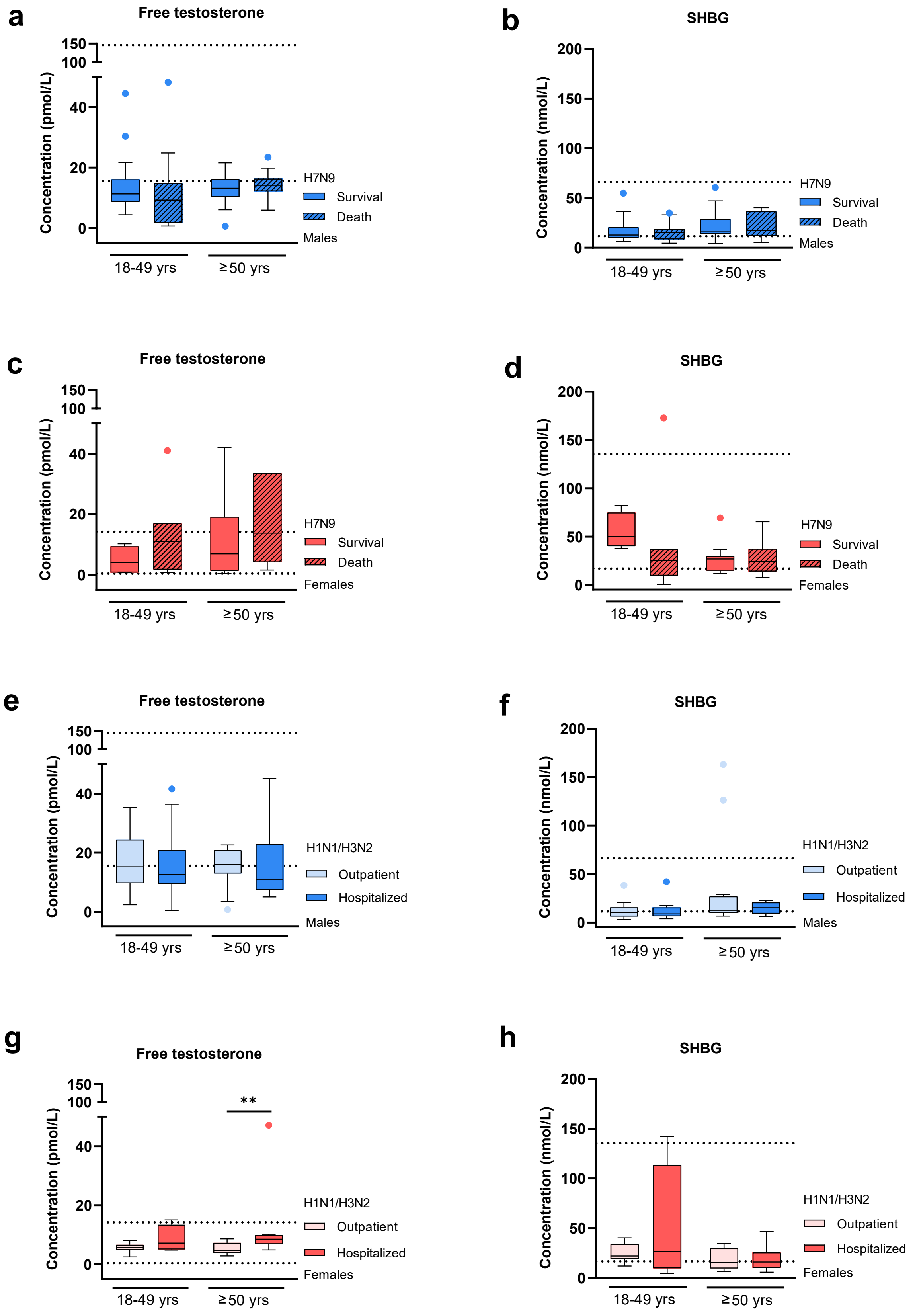
a
Free testosterone
Concentration (pmol/L)
150
100
40
20
0
H7N9
Survival
Death
Males
18-49 yrs
≥50 yrs
b
SHBG
Concentration (nmol/L)
200
150
100
50
0
H7N9
Survival
Death
Males
18-49 yrs
≥50 yrs
c
Free testosterone
Concentration (pmol/L)
150
100
40
20
0
H7N9
Survival
Death
Females
18-49 yrs
≥50 yrs
d
SHBG
Concentration (nmol/L)
200
150
100
50
0
H7N9
Survival
Death
Females
18-49 yrs
≥50 yrs
e
Free testosterone
Concentration (pmol/L)
150
100
40
20
0
H1N1/H3N2
Outpatient
Hospitalized
Males
18-49 yrs
≥50 yrs
f
SHBG
Concentration (nmol/L)
200
150
100
50
0
H1N1/H3N2
Outpatient
Hospitalized
Males
18-49 yrs
≥50 yrs
g
Free testosterone
**
Concentration (pmol/L)
150
100
40
20
0
H1N1/H3N2
Outpatient
Hospitalized
Females
18-49 yrs
≥50 yrs
h
SHBG
Concentration (nmol/L)
200
150
100
50
0
H1N1/H3N2
Outpatient
Hospitalized
Females
18-49 yrs
≥50 yrs

Supplementary Figure 1: Free testosterone and SHBG levels in H7N9 and seasonal influenza cases

(a, b) Shown are the free testosterone and sex hormone binding globulin (SHBG) levels from H7N9-infected male patients in dependency of disease outcome in each age group (18-49 yrs survival/death: n=18/15, ≥50 yrs survival/death: n=27/11); (c, d) Shown are the free testosterone and SHBG levels from H7N9-infected female patients in dependency of disease outcome in each age group (18-49 yrs survival/death: n=4/7, ≥50 yrs survival/death: n=10/6); (e, f) Shown are the free testosterone (18-49 yrs outpatient/hospitalized: n=15/11, ≥50 yrs outpatient/hospitalized: n=12/14) and SHBG levels (18-49 yrs outpatient/hospitalized: n=16/11, ≥50 yrs outpatient/hospitalized: n=12/14) from seasonal influenza-infected male patients in both age groups; (g, h) Shown are the free testosterone and SHBG levels from seasonal influenza-infected female patients in both age groups (18-49 yrs outpatient/hospitalized: n=12/4, ≥50 yrs n=13/10). (a-g) Data are presented as Box-and-whisker plots (Tukey). The horizontal line in each box represents the median value. The 25th-75th percentiles represent the endpoints of the box. The whiskers stretch to the lowest and highest values within 1.5 times the interquartile range (IQR) from the 25th-75th percentiles. Dots represent outliers according to Tukey's definition. The two dotted lines in each figure represent the reference ranges for free testosterone (a, e: 15.6-145.6 (pmol/L); c, g: 0.35-14.21 (pmol/L)) and SHBG (b, f: 11.5-66.3 (nmol/L); d, h: 16.8-135.5 (nmol/L)). Statistically significant differences between groups were determined using unpaired, two-tailed non-parametric analysis (Mann-Whitney test), and a *P* value of < 0.05 was considered significant (* $p < 0.05$, ** $p < 0.01$, *** $p < 0.001$, **** $p < 0.0001$). Source data are provided as Source Data File.

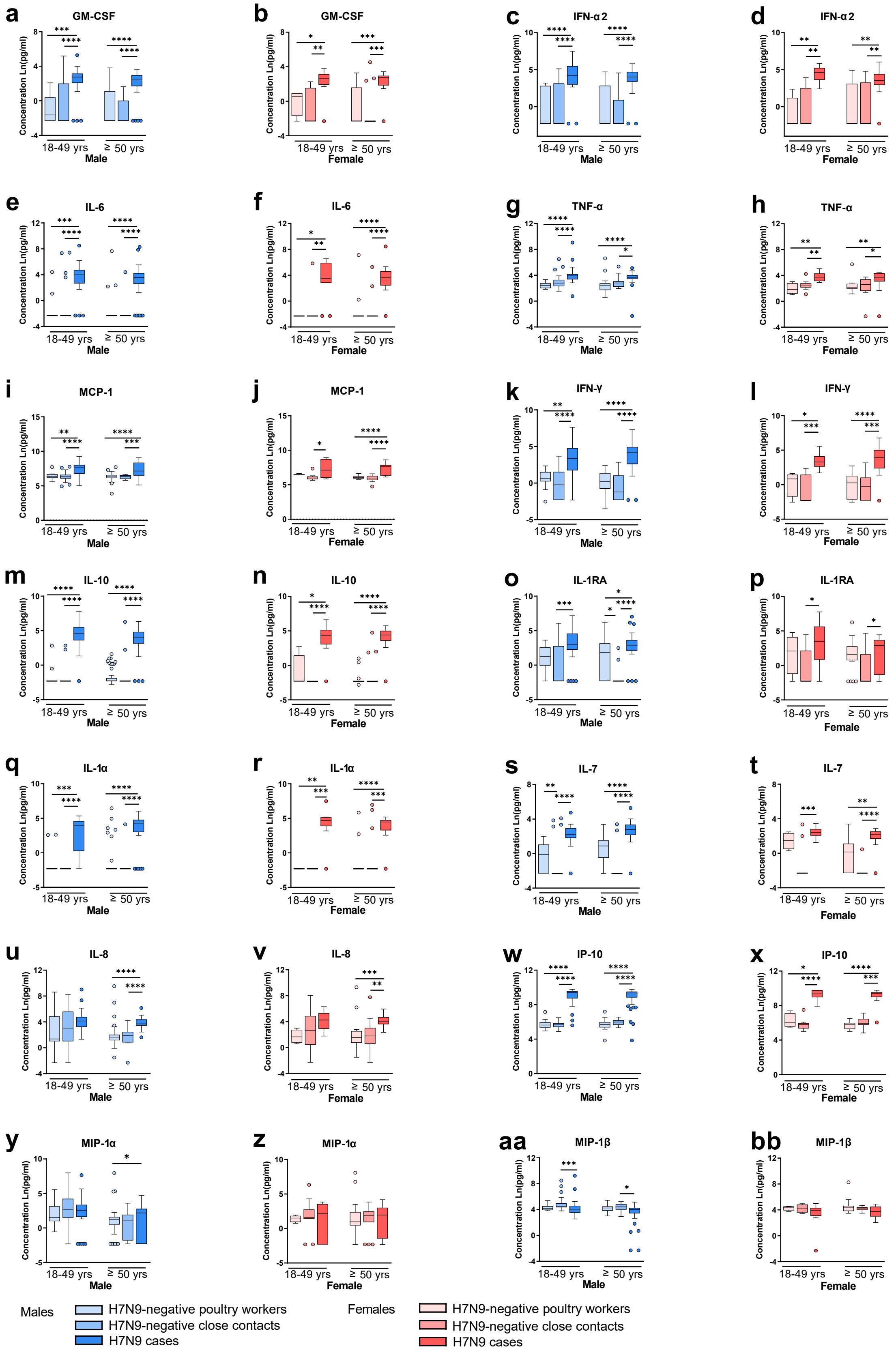

a GM-CSF
b GM-CSF
c IFN-α2
d IFN-α2
e IL-6
f IL-6
g TNF-α
h TNF-α
i MCP-1
j MCP-1
k IFN-γ
l IFN-γ
m IL-10
n IL-10
o IL-1RA
p IL-1RA
q IL-1α
r IL-1α
s IL-7
t IL-7
u IL-8
v IL-8
w IP-10
x IP-10
y MIP-1α
z MIP-1α
aa MIP-1β
bb MIP-1β
Concentration Ln(pg/ml)
18-49 yrs
≥ 50 yrs
Male
Female
Males
H7N9-negative poultry workers
H7N9-negative close contacts
H7N9 cases
Females
H7N9-negative poultry workers
H7N9-negative close contacts
H7N9 cases

Supplementary Figure 2:

Cytokine and chemokine response in H7N9 IAV-infected patients compared to control groups

(a-bb) Shown are the expression levels of cytokines and chemokines in the sera from H7N9 IAV-infected male and female patients who were significantly altered compared to control groups, each divided into two groups based on age: 18-49 years (yrs) and ≥ 50 years old. The number of samples in males were as follows: poultry workers (18-49 yrs: n=12, ≥50 yrs: n=45), H7N9 close contacts (18-49 yrs: n=29, ≥50 yrs: n=16), and H7N9 cases (18-49 yrs: n=33, ≥50 yrs: n=38). The number of samples in females were as follows: poultry workers (18-49 yrs: n=4, ≥50 yrs: n=20), H7N9 close contacts (18-49 yrs: n=11, ≥50 yrs: n=15), and H7N9 cases (18-49 yrs: n=10, ≥50 yrs: n=16). Cytokine/chemokine expression values were used after Ln (*natural logarithm*) transformation. The measurement was carried out using a multiplex immunoassay. The following analytes were included: GM-CSF (granulocyte-macrophage colony-stimulating factor; a, b), IFN-α2 (interferon alpha 2; c, d), IL-6 (interleukin 6; e, f), TNF-α (tumor necrosis factor alpha; g, h), MCP-1/CCL2 (monocyte chemoattractant protein 1; i, j), IFN-γ (interferon gamma; k, l), IL-10 (interleukin-10; m, n), IL-1RA (interleukin 1 receptor antagonist; o, p), IL-1α (interleukin 1 alpha; q, r), IL-7 (interleukin 7; s, t), IL-8 (interleukin 8; u, v), IP10/CXL10 (interferon gamma-induced protein 10; w, x), MIP-1β/CCL4 (macrophage inflammatory protein 1 beta; y, z) and MIP-1α/CCL3 (macrophage inflammatory protein 1 alpha; aa, bb). Data are presented as Box-and-whisker plots (Tukey). The horizontal line in each box represents the median value. The 25th-75th percentiles represent the endpoints of the box. The whiskers stretch to the lowest and highest values within 1.5 times the interquartile range (IQR) from the 25th-75th percentiles. Dots represent outliers according to Tukey's definition. Statistically significant differences between groups were determined using the Kruskal-Wallis test and Dunn's post hoc test. A P value of < 0.05 was considered a significant difference (* $p < 0.05$, ** $p < 0.01$, *** $p < 0.001$, **** $p < 0.0001$). Source data are provided as Source Data File.

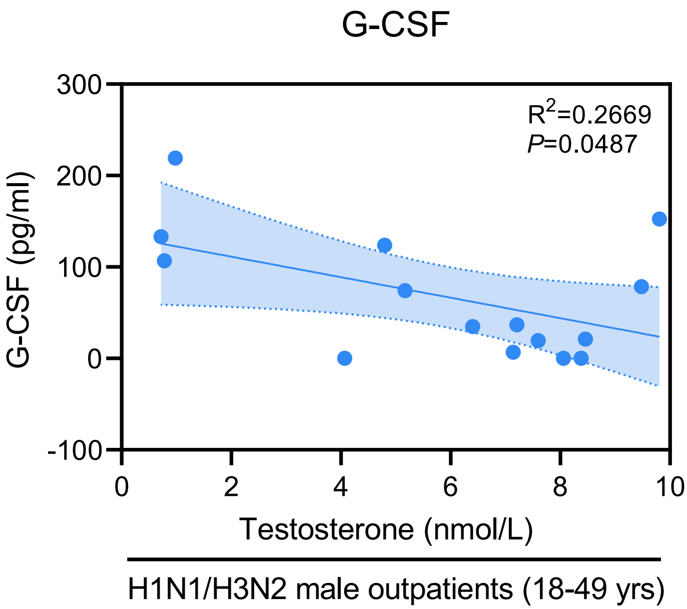
G-CSF
G-CSF (pg/ml)
300
200
100
0
-100
R2=0.2669
P=0.0487
0
2
4
6
8
10
Testosterone (nmol/L)
H1N1/H3N2 male outpatients (18-49 yrs)

Supplementary Figure 3:

Linear regression analysis of testosterone-modulated inflammatory immune responses in seasonal influenza-infected males

Shown is the observed regression between testosterone and G-CSF (granulocyte colony-stimulating factor) in seasonal influenza male outpatients aged 18-49 years (n=15). Testosterone expression levels measured in the plasma from seasonal IAV-infected male outpatients were plotted over the respective G-CSF levels. Two-tailed linear regression analysis was performed from outpatients and hospitalized patients, and no adjustment for multiple hypotheses was performed due to the explorative nature of the study. The best-fit line with 95% confidence intervals is shown. The measure of centre for the error bands is the regression line. The R squared and *P* values are shown in the graph. Source data are provided as Source Data File.

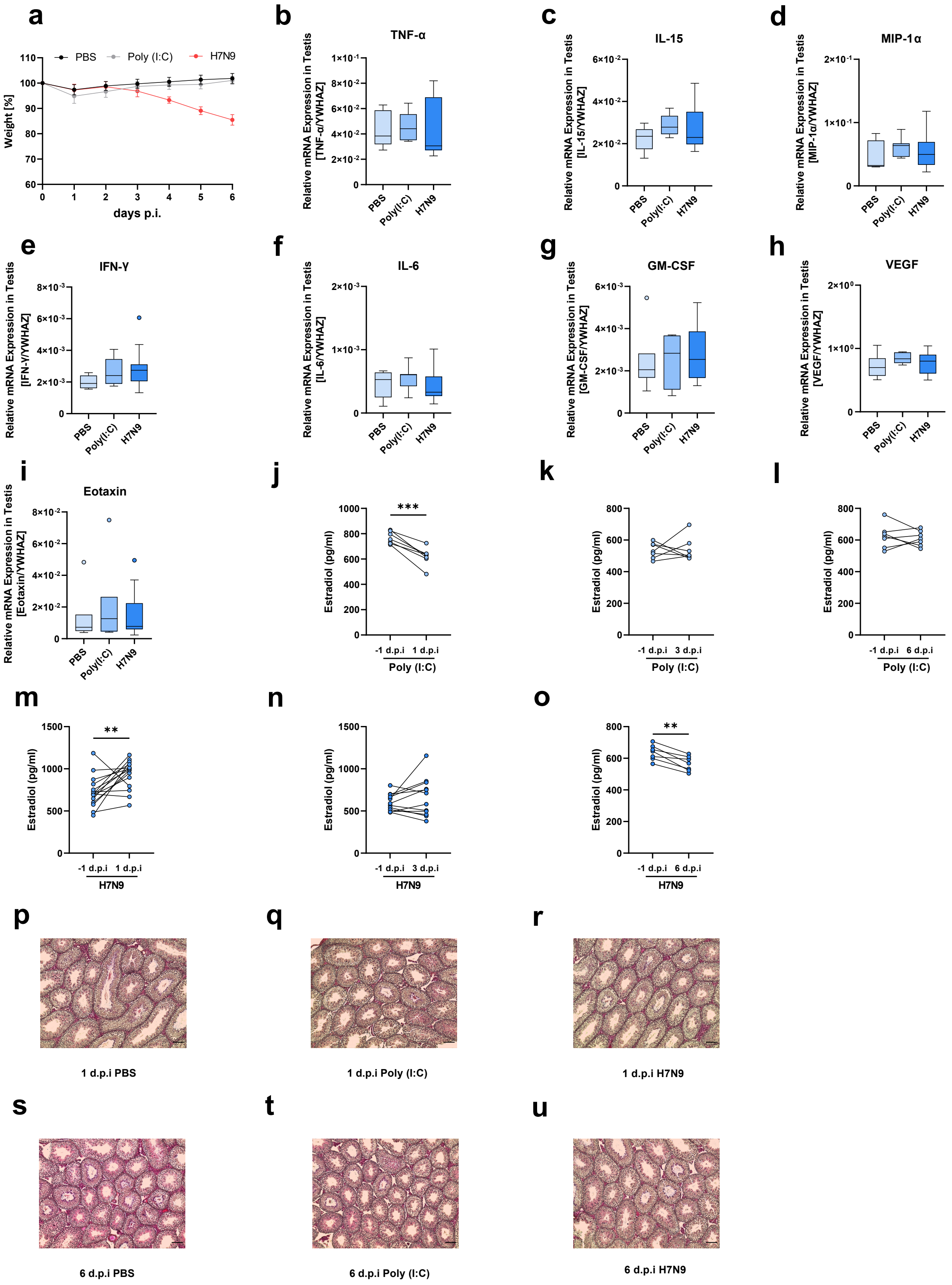
a
PBS
Poly (I:C)
H7N9
Weight [%]
days p.i.
b
TNF-α
Relative mRNA Expression in Testis [TNF-α/YWHAZ]
c
IL-15
Relative mRNA Expression in Testis [IL-15/YWHAZ]
d
MIP-1α
Relative mRNA Expression in Testis [MIP-1α/YWHAZ]
e
IFN-γ
Relative mRNA Expression in Testis [IFN-γ/YWHAZ]
f
IL-6
Relative mRNA Expression in Testis [IL-6/YWHAZ]
g
GM-CSF
Relative mRNA Expression in Testis [GM-CSF/YWHAZ]
h
VEGF
Relative mRNA Expression in Testis [VEGF/YWHAZ]
i
Eotaxin
Relative mRNA Expression in Testis [Eotaxin/YWHAZ]
PBS
Poly(I:C)
H7N9
j
k
l
Estradiol (pg/ml)
-1 d.p.i
1 d.p.i
3 d.p.i
6 d.p.i
Poly (I:C)
m
n
o
H7N9
p
q
r
1 d.p.i PBS
1 d.p.i Poly (I:C)
1 d.p.i H7N9
s
t
u
6 d.p.i PBS
6 d.p.i Poly (I:C)
6 d.p.i H7N9

Supplementary Figure 4:

Weight loss, testicular inflammation, histopathology and estradiol levels in H7N9 infected mice

(a) Weight loss of PBS-, Poly (I:C)- and H7N9-treated mice up to 6 d.p.i (b-i) Cytokine and chemokine levels without significant differences in the testes from PBS (n=7)-, Poly (I:C)-(n=7)- and H7N9 (n=16)-treated mice at 3 d.p.i. Data are presented as Box-and-whisker plots (Tukey). The horizontal line in each box represents the median value. The 25th-75th percentiles represent the endpoints of the box. The whiskers stretch to the lowest and highest values within 1.5 times the interquartile range (IQR) from the 25th-75th percentiles. Dots represent outliers according to Tukey's definition. (j-l) Estradiol levels in Poly (I:C)-treated mice at 1 day before treatment (-1 d.p.i) vs. treated mice at 1 day (n=7 vs. n=7), 3 days (n=7 vs. n=7) and 6 days (n=7 vs. n=7) post treatment. (m-o) Estradiol levels in H7N9-infected mice at 1 day before infection (-1 d.p.i) vs. infected mice at 1 day (n=15 vs. n=15), 3 days (n=12 vs. n=12) and 6 days post infection (n=7 vs. n=7). (p-u) Hematoxylin and eosin (HE)-stained paraffin sections from Bouin fixed testis tissue at 1 (p-r) and 6 d.p.i (s-u) were evaluated via light microscopy. Shown are representative histological images of the testes of PBS (1 d.p.i., n=6; 6 d.p.i., n=7)- or Poly (I:C) (1 d.p.i., n=6; 6 d.p.i., n=7)-control treated and H7N9-infected (1 d.p.i., n=6; 6 d.p.i., n=7) mice (10X). Scale bars (100 µm) are shown in the bottom right of each micrograph. (b-i) Statistically significant differences among the three groups were determined using either two-tailed one-way ANOVA or the Kruskal-Wallis test (b-i). (j-o) Statistically significant differences in estradiol were determined by paired, two-tailed *t test* or non-parametric analysis (j-o). A P value of < 0.05 was considered a significant difference (* $p < 0.05$, ** $p < 0.01$, *** $p < 0.001$, **** $p < 0.0001$). Source data are provided as Source Data File.

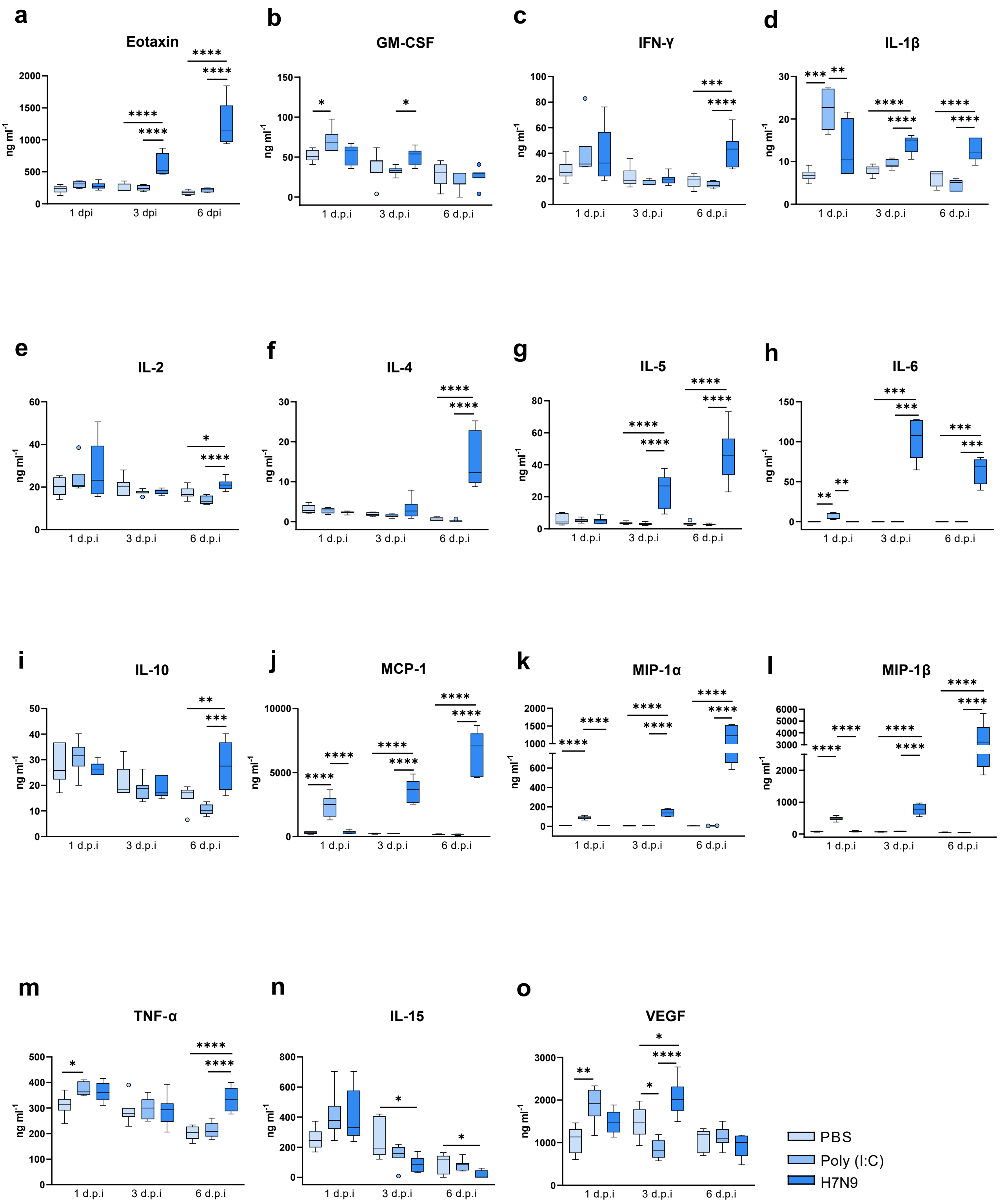
a
Eotaxin
b
GM-CSF
c
IFN-Ɣ
d
IL-1β
e
IL-2
f
IL-4
g
IL-5
h
IL-6
i
IL-10
j
MCP-1
k
MIP-1α
l
MIP-1β
m
TNF-α
n
IL-15
o
VEGF
ng ml-1
1 dpi
3 dpi
6 dpi
1 d.p.i
3 d.p.i
6 d.p.i
PBS
Poly (I:C)
H7N9

Supplementary Figure 5:

Pulmonary inflammatory response in H7N9-infected mice

(a-o) Cytokines and chemokines were measured in the lungs of PBS and Poly (I:C) control-treated or H7N9 infected mice at 1d.p.i (PBS: n=6, Poly (I:C): n=6, H7N9:n=6), 3 d.p.i (PBS: n=7, Poly (I:C):n=7, H7N9: n=7) and 6 d.p.i (PBS: n=7, Poly (I:C): n=7, H7N9:n=using a Bio-Plex Pro Mouse Cytokine multiplex assay. The following analytes were included: Eotaxin (a), GM-CSF (Granulocyte-macrophage colony-stimulating factor; b), IFN-γ (interferon gamma; c), IL-1β (interleukin 1 beta; d), IL-2 (interleukin 2; e), IL-4 (interleukin 4; f), IL-5 (interleukin 5; g), IL-6 (interleukin 6; h), IL-10 (interleukin 10; i), MCP-1 (monocyte chemoattractant protein 1; j), MIP-1α (macrophage inflammatory protein 1 alpha; k), MIP-1β (macrophage inflammatory protein 1 beta; l), TNF-α (tumor necrosis factor alpha; m), IL-15 (interleukin 15; n), and VEGF (vascular epidermal growth factor; o). Data are presented as box-and-whisker plots (Tukey). The horizontal line in each box represents the median value. The 25th-75th percentiles represent the endpoints of the box. The whiskers stretch to the lowest and highest values within 1.5 times the interquartile range (IQR) from the 25th-75th percentiles. Dots represent outliers according to Tukey's definition. Statistically significant differences among groups were determined using either two-tailed ANOVA or the Kruskal-Wallis test. A *P* value of < 0.05 was considered a significant difference (* $p < 0.05$, ** $p < 0.01$, *** $p < 0.001$, **** $p < 0.0001$). Source data are provided as Source Data File.

Supplementary Tables

Supplementary Table 1. qRT-PCR primers for murine cytokines/chemokines and the host gene used in this study

Gene	Sequence 5'-3'
TNF-α	Forward: CAGAAAGCATGATCCGCGAC
	Reverse: GGCCATAGAACTGATGAGAGGG
MIP-1α	Forward: CAGCCAGGTGTCATTTTCCTG
	Reverse: CTCGATGTGGCTACTTGGCA
MIP-1β	Forward: AACCTAACCCCGAGCAACAC
	Reverse: GGGTCAGAGCCCATTGGTG
IFN-γ	Forward: AGGTCAACAACCCACAGGTC
	Reverse: GAATCAGCAGCGACTCCTTT
IL-1β	Forward: GAGCCCATCCTCTGTGACTC
	Reverse: AGCTCATATGGGTCCGACAG
IL-10	Forward: GGTTGCCAAGCCTTATCGGA
	Reverse: CACCTTGGTCTTGGAGCTTATT
IL-6	Forward: CTCCCAACAGACCTGTCTATAC
	Reverse: GTGCATCATCGTTGTTCATAC
Eotaxin	Forward: GAGCTCCACAGCGCTTCTAT
	Reverse: GAAGTTGGGATGGAGCCTGG
VEGF	Forward: TCTGAGAGAGGCCGAAGTCC
	Reverse: GCGGGGTGCTTTTGTAGACT
IL-15	Forward: CGCCCAAAAGACTTGCAGTG
	Reverse: GGTGGATTCTTTCCTGACCTCT
GM-CSF	Forward: AAGGTCCTGAGGAGGATGTGG
	Reverse: GTCTGCACACATGTTAGCTTCTTG
YWHAZ	Forward: CACGCTCCCTAACCTTGCTT
	Reverse: ATCGTAGAAGCCTGACGTGG

Supplementary Table 2. Additional characteristics of H7N9 cases

Characteristic	18-49 years (*n*=44)	≥50 years (*n*=54)
Time from illness onset to sampling (days)		
Median	6.5	7.5
Q1-Q3	5-8.3	6-9
Antiviral treatment (%)		
Yes	39 (89%)	46 (85%)
No	5 (11%)	8 (15%)
Year (%)		
2014-2016	10 (23%)	10 (19%)
2017	34 (77%)	44 (81%)
Region (%)		
South	32 (73%)	33 (61%)
North	12 (27%)	21 (39%)

Data are median (25th quartile-75th quartile, Q1-Q3) or *n* (%).
