# Supplementary material for: H7N9 avian influenza virus infection in men is associated with testosterone depletion
Source: Nat Commun. 2022 Nov 14;13:6936. doi: 10.1038/s41467-022-34500-5 (PMC9662777; doi:10.1038/s41467-022-34500-5)
Supplement: Supplementary file 3 — Reporting Summary [file 41467_2022_34500_MOESM3_ESM.pdf]

nature portfolio

Corresponding author(s): Yuelong Shu, Gülsah Gabriel

Last updated by author(s): Oct 24, 2022

Reporting Summary

Nature Portfolio wishes to improve the reproducibility of the work that we publish. This form provides structure for consistency and transparency in reporting. For further information on Nature Portfolio policies, see our Editorial Policies and the Editorial Policy Checklist.

Statistics

For all statistical analyses, confirm that the following items are present in the figure legend, table legend, main text, or Methods section.

n/a	Confirmed	
☐	☒	The exact sample size (*n*) for each experimental group/condition, given as a discrete number and unit of measurement
☐	☒	A statement on whether measurements were taken from distinct samples or whether the same sample was measured repeatedly
☐	☒	The statistical test(s) used AND whether they are one- or two-sided<br>*Only common tests should be described solely by name; describe more complex techniques in the Methods section.*
☐	☒	A description of all covariates tested
☐	☒	A description of any assumptions or corrections, such as tests of normality and adjustment for multiple comparisons
☐	☒	A full description of the statistical parameters including central tendency (e.g. means) or other basic estimates (e.g. regression coefficient) AND variation (e.g. standard deviation) or associated estimates of uncertainty (e.g. confidence intervals)
☐	☒	For null hypothesis testing, the test statistic (e.g. *F*, *t*, *r*) with confidence intervals, effect sizes, degrees of freedom and *P* value noted<br>*Give P values as exact values whenever suitable.*
☒	☐	For Bayesian analysis, information on the choice of priors and Markov chain Monte Carlo settings
☒	☐	For hierarchical and complex designs, identification of the appropriate level for tests and full reporting of outcomes
☒	☐	Estimates of effect sizes (e.g. Cohen's *d*, Pearson's *r*), indicating how they were calculated

Our web collection on statistics for biologists contains articles on many of the points above.

Software and code

Policy information about availability of computer code

Data collection	Excel 2016 was used for data collection in this study.
Data analysis	GraphPad Prism 9.0.1 (GraphPad Software, Inc.), SAS®, version 9.4 TS level 1M5 (SAS Institute Inc., Cary, NC, United States), IBM® SPSS® Statistics 27, and G Power (Version 3.1) were used for data analysis or planing of the animal experiment in this study.<br>The code of SAS®, version 9.4 TS level 1M5 (SAS Institute Inc., Cary, NC, United States) is used including secured Macro-codes developed by the University of Veterinary Medicine Hannover (TiHo). Therefore, code is available for interested users by request only.

For manuscripts utilizing custom algorithms or software that are central to the research but not yet described in published literature, software must be made available to editors and reviewers. We strongly encourage code deposition in a community repository (e.g. GitHub). See the Nature Portfolio guidelines for submitting code & software for further information.

Data

Policy information about availability of data

All manuscripts must include a data availability statement. This statement should provide the following information, where applicable:

- Accession codes, unique identifiers, or web links for publicly available datasets
- A description of any restrictions on data availability
- For clinical datasets or third party data, please ensure that the statement adheres to our policy

All relevant data are available within the paper or its supplementary information files. Source data are provided with this paper. The dataset is limited to academic

use.

Human research participants

Policy information about studies involving human research participants and Sex and Gender in Research.

Reporting on sex and gender	This study aimed to explore the association of biological factors and male-biased infection of H7N9 influenza virus. Hence, sex is an important variate that is considered in our study design. This information was obtained during sample collection through the National Influenza Surveillance Network in local Centers for Disease Control and Prevention (CDCs), China. Informed consent has been obtained. Overall, a total of 369 study subjects with complete epidemiology information (including sex) were included in this study. Analysis of biological factors (sex hormones, cytokines) of interest was performed in dependency of sex in this study.
Population characteristics	Sex and age were obtained from all study subjects. Study subjects (i.e. H7N9 patients and control groups) were divided into two age groups, that is 18 to 49 years and ≥ 50 years of age. Within the younger age group, 121 study subjects were male and 49 were female. Within the older age group, 125 study subjects were male and 74 were female.<br>In addition, for H7N9 patients, information on the illness onset date, sample collection date, final disease outcome and antiviral treatment were obtained as well (presented as supplementary table).
Recruitment	We used secondary data from archived blood samples of the study groups of interest. Therefore, no recruitment was conducted from the field in this study. Patients with complete epidemiological information and required amount of sample volume for analysis were included. Healthy controls were included by matching age or sex. By the nature of the study, multiple adjustments with other factors influencing sex hormones were therefore not taken into account. Our data is obtained from the Influenza Surveillance Network in the local Center for Disease Control and Prevention (CDC), China, which is responsible for identification and reporting all novel influenza cases nationwide. However, as all systems with a voluntary participation, this is never free from any selection bias.
Ethics oversight	Sampling from laboratory-confirmed H7N9 avian influenza cases, seasonal influenza cases, close contacts of H7N9 cases and poultry workers was reviewed and approved by the Ethics Committee of the National Institute for Viral Disease Control and Prevention, China CDC. Informed consent was obtained from all subjects in this study.

Note that full information on the approval of the study protocol must also be provided in the manuscript.

Field-specific reporting

Please select the one below that is the best fit for your research. If you are not sure, read the appropriate sections before making your selection.

☒ Life sciences ☐ Behavioural & social sciences ☐ Ecological, evolutionary & environmental sciences

For a reference copy of the document with all sections, see nature.com/documents/nr-reporting-summary-flat.pdf

Life sciences study design

All studies must disclose on these points even when the disclosure is negative.

Sample size	As this is a retrospective observational study, within the human cohort we used previously stored serum or plasma samples from study participants. Hence, no sample-size calculation was performed. We first selected the available sample size for H7N9 cases with complete epidemiological information and required volume of serum or plasma for subsequent measurements. Control groups were selected by matching age or sex to the group of "H7N9 cases" if available. Archived plasma samples from seasonal influenza cases were selected based on the same age criteria.<br>The group sizes used for the animal experiment were based on the study-specific animal project license. Therefore, group sizes reflect ethical and biometric considerations to allow for experiments to provide sufficient data for statistical analysis. In this study, a minimum group size of 7 was determined based on pre-existing data and using the the software G Power. The infection with H7N9 was performed twice and data were merged to have sufficient power for statistical analysis. This was necessary due to a natural high variation in testosterone levels in young male mice and the exclusion of statistical outliers with very high testosterone levels ('alpha males') from downstream analyses. Control inoculation with PBS and Poly I:C was only performed once due to a limited number of animals available for this study. Since only male mice were used in this study, no sex-stratification of the data was performed.
Data exclusions	Within the human cohort, no data or outliers were excluded. In molecular assessment of animal data, we excluded outliers that were detected using GraphPad Prism 9.0.1 (GraphPad Software, Inc.) software.
Replication	In the human cohort (Fig. 1-5, Supplementary Fig. 1-3), sex hormones were measured only once without technical replicates due to the high amount of volume required for each measurement. Cytokine and chemokine concentrations were measured in technical duplicates. None of the measurements can be repeated as there are no remaining serum or plasma samples available.<br><br>The animal experiment (Fig. 6, Supplementary Fig. 4 and 5) was performed once for PBS or Poly I:C control inoculation, and twice for H7N9 infection. Data from both experiments were merged. Due to the explorative nature of this study, only a limited number of animals was approved by the authorities. Thus, for ethical reasons these experiments cannot be repeated. Plasma sex hormone levels, virus titers and cytokine expression in the lung as well as viral RNA levels and cytokine expression in the testis were determined with a biological replicate of n

	= 7 (PBS, Poly I:C) or n= 17 (H7N9), without technical replicates. Histological analysis of the testis was performed with a biological replicate of n=6-7, and representative images are shown. All measurements involving qRT-PCR were performed in technical duplicates.<br><br>All attempts at replication were successful.
Randomization	Randomization is not applicable for our retrospective study in the human cohort. Firstly, we used stored serum or plasma samples collected from the National Influenza Surveillance Network in China CDC once there were confirmed H7N9 or seasonal influenza cases. Therefore, it is not possible to conduct sampling randomly. Secondly, the sample size of the available study subjects (which must have complete epidemiology information and required amount of sample for measurement in this study) might not be large enough for randomization. Randomization cannot guarantee equivalence among groups when the sample size in each group is small and that a small sample may result in an underpowered test.<br><br>In the animal experiment, mice were grouped randomly after delivery from the supplier.
Blinding	We performed a retrospective study in the human cohort by using archived serum or plasma samples collected from the National Influenza Surveillance Network in China CDC once there were confirmed H7N9 or seasonal influenza cases. Therefore blinding was not applicable for sample collection. Blinding was applied for data analysis within the human cohort.<br><br>No blinding was performed when using infectious animals or samples due to biosafety regulations. However, blinding was performed when handling and assessing non-infectious material (e.g. fixed testis tissue for histological evaluation).

Reporting for specific materials, systems and methods

We require information from authors about some types of materials, experimental systems and methods used in many studies. Here, indicate whether each material, system or method listed is relevant to your study. If you are not sure if a list item applies to your research, read the appropriate section before selecting a response.

Materials & experimental systems

n/a	Involved in the study	
☐	☒	Antibodies
☐	☒	Eukaryotic cell lines
☒	☐	Palaeontology and archaeology
☐	☒	Animals and other organisms
☒	☐	Clinical data
☒	☐	Dual use research of concern

Methods

n/a	Involved in the study	
☒	☐	ChIP-seq
☒	☐	Flow cytometry
☒	☐	MRI-based neuroimaging

Antibodies

Antibodies used	Bead-coupled antibodies against EGF, Eotaxin, G-CSF, GM-CSF, IFN-α2, IFN-γ, IL-10, IL12-P40, IL12-P70, IL-13, IL-15, IL-17A, IL-1RA, IL-1α, IL-1β, IL-2, IL-3, IL-4, IL-5, IL-6, IL-7, IL-8, IP10, MCP-1, MIP-1α, MIP-1β, TNF-α, TNF-β, VEGF as part of the Luminex bead based multiplex assay (HCYTOMAG-60K, Millipore, USA) used to measure cytokines and chemokines in human samples. Bead-coupled antibodies against Eotaxin, G-CSF, GM-CSF, IFN-γ, IL-1β, IL-2, IL-3, IL-4, IL-5, IL-6, IL-10, MCP-1, MIP-1α, MIP-1β, TNF-α, IL-15, and VEGF as part of the Bio-Plex Pro Mouse Cytokine kits (Bio-rad) used to measure cytokines and chemokines in mice lung tissues. Antibodies against estradiol, testosterone, free testosterone and SHBG as part of the BECKMAN COULTER Access Immunoassay Systems or ELISA KITs to measure sex hormones in this study.
Validation	Antibodies/Assay systems are commercially available.

Eukaryotic cell lines

Policy information about cell lines and Sex and Gender in Research

Cell line source(s)	Madin-Darby Canine Kidney (MDCK II) (♀) ATCC (CCL-34)
Authentication	No authentication was performed after receiving the cells from the supplier.
Mycoplasma contamination	The eukaryotic cell line used in this study were regularly verified to be negative for mycoplasma spp contamination using the Venor®GeM Classic Mycoplasma PCR Detection Kit (Minerva Biolabs GmbH) according to manufacturer's instructions. No contamination of Mycoplasma was detected.
Commonly misidentified lines (See ICLAC register)	No commonly misidentified cell lines were used.

Animals and other research organisms

Policy information about studies involving animals; ARRIVE guidelines recommended for reporting animal research, and Sex and Gender in Research

Laboratory animals	Mouse (Mus musculus), C57BL/6J, male, 10 weeks old at experimental start.
Wild animals	No wild animals were involved in this study.
Reporting on sex	Only male mice were used in this study to further evaluate a potential causal link of H7N9 infection and testosterone depletion as seen in H7N9 patients within the human cohort.
Field-collected samples	No field collected samples were used in this study.
Ethics oversight	All animal experiments were performed in strict accordance with the German animal protection law (Behörde für Gesundheit und Verbraucherschutz, Hamburg, Germany; licensing number: N124/2021).

Note that full information on the approval of the study protocol must also be provided in the manuscript.
